# (Acetyl­acetonato-κ^2^ *O*,*O*′)aqua­[2-(2-nitro­phen­oxy)-*N*′-(2-oxidobenzyl­idene-κ*O*)acetohydrazidato-κ^2^ *O*,*N*′]manganese(III)

**DOI:** 10.1107/S1600536809028177

**Published:** 2009-07-25

**Authors:** Zi-Jing Xiao

**Affiliations:** a College of Materials Science & Engineering, Huaqiao University, Quanzhou 362021, People’s Republic of China

## Abstract

In the title complex, [Mn(C_15_H_11_N_3_O_5_)(C_5_H_7_O_2_)(H_2_O)], the Mn^III^ ion has a distorted octa­hedral coordination geometry. It is coordinated by a phen­oxy O atom, a hydrazine N atom and a carbonyl O atom of the 2-(2-nitro­phen­oxy)-*N*′-(2-oxidobenzyl­idene-κ*O*)acetohydrazidate dianion, by two O atoms of the acetyl­acetonate anion and by the O atom of a coordinated water mol­ecule. In the crystal structure, complex mol­ecules are linked into centrosymmetric dimeric units through four inter­molecular O—H⋯O hydrogen bonds involving both H atoms of the coordinated water mol­ecule.

## Related literature

For the biological activity and chemical versatility of hydrazone complexes, see: Liu & Gao (1998 ▶); Iskander *et al.* (2001 ▶); Cariati *et al.* (2002 ▶); Sreekanth *et al.* (2004 ▶); Bai *et al.* (2006 ▶); Mondal *et al.* (2008 ▶). For phenoxy­acetyl­hydrazone complexes, see: Chen & Liu (2004 ▶); Sun *et al.* (2005 ▶); Chen & Liu (2006 ▶).
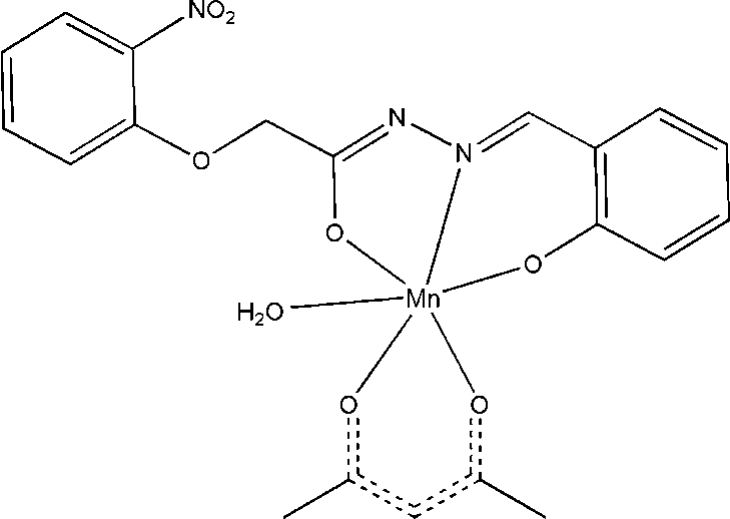


## Experimental

### 

#### Crystal data

[Mn(C_15_H_11_N_3_O_5_)(C_5_H_7_O_2_)(H_2_O)]
*M* *_r_* = 485.33
Monoclinic, 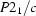

*a* = 8.5217 (6) Å
*b* = 13.9263 (10) Å
*c* = 17.6311 (10) Å
β = 92.725 (3)°
*V* = 2090.0 (2) Å^3^
*Z* = 4
Mo *K*α radiation
μ = 0.69 mm^−1^
*T* = 293 K
0.58 × 0.32 × 0.27 mm

#### Data collection

Rigaku R-AXIS RAPID Imaging Plate diffractometer
Absorption correction: multi-scan (*TEXRAY*; Molecular Structure Corporation, 1999 ▶) *T*_min_ = 0.766, *T*_max_ = 0.832
4921 measured reflections
4735 independent reflections
3155 reflections with *I* > 2σ(*I*)
*R*_int_ = 0.046

#### Refinement

*R*[*F* ^2^ > 2σ(*F* ^2^)] = 0.038
*wR*(*F* ^2^) = 0.086
*S* = 0.88
4735 reflections
293 parameters
2 restraints
H-atom parameters constrained
Δρ_max_ = 0.41 e Å^−3^
Δρ_min_ = −0.26 e Å^−3^

### 

Data collection: *TEXRAY* (Molecular Structure Corporation, 1999 ▶); cell refinement: *TEXRAY*; data reduction: *TEXSAN* (Molecular Structure Corporation, 1999 ▶); program(s) used to solve structure: *SHELXS98* (Sheldrick, 2008 ▶); program(s) used to refine structure: *SHELXL98* (Sheldrick, 2008 ▶); molecular graphics: *ORTEX* (McArdle, 1995 ▶); software used to prepare material for publication: *SHELXL97*.

## Supplementary Material

Crystal structure: contains datablocks I, global. DOI: 10.1107/S1600536809028177/fj2221sup1.cif

Structure factors: contains datablocks I. DOI: 10.1107/S1600536809028177/fj2221Isup2.hkl

Additional supplementary materials:  crystallographic information; 3D view; checkCIF report

## Figures and Tables

**Table 1:** Hydrogen-bond geometry (Å, °)

*D*—H⋯*A*	*D*—H	H⋯*A*	*D*⋯*A*	*D*—H⋯*A*
O1*W*—H01⋯O4^i^	0.88	2.16	2.955 (2)	150
O1*W*—H02⋯O2^i^	0.88	1.96	2.829 (2)	169

Symmetry code: (i) 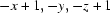.

**Fig. 1.**
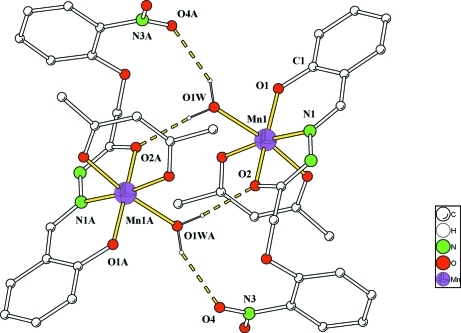
The molecular structure of (I), showing 20% probability displacement ellipsoids and the atom numbering scheme.

**Fig. 2.**
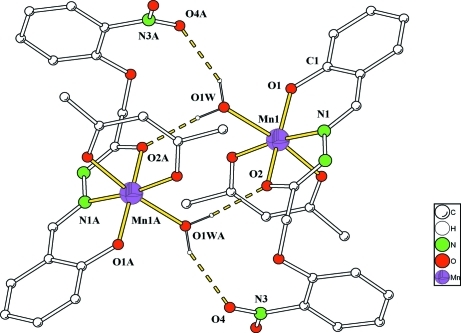
Extended centrosymmetrical dimer structure of (I).

## supplementary crystallographic information

### Comment

The design and construction of hydrazone complexes are of great interest due to their various structures and their biological activities and chemical versatility (Liu & Gao, 1998; Iskander *et al.*, 2001; Cariati *et al.*, 2002; Sreekanth *et al.*, 2004; Bai *et al.*, 2006; Mondal *et al.*, 2008). Relatively speaking, only a few crystal structures of phenoxyacetylhydrazone complexes have been studied (Chen & Liu, 2004; Sun *et al.*, 2005; Chen & Liu, 2006).

As shown in Fig. 1, the Mn^III^ ion in (I) is octahedrally coordinated by phenol atom O1, hydrazine atom N1 and carbonyl atom O2 from the phenoxyacetylhydrazone ligand *L*^2-^, two oxygen atoms (O6 and O7) from an acac^-^ ligand, and O1W atom from coordinated water molecule. Atoms O1, N1, O2 and O7 form the equatorial plane, while the atoms O6 and O1W occupy the two axial positions. The bond distance of Mn1—O6(acac^-^) (2.130 (2) Å) is much longer than the one of Mn1—O7(acac^-^) (1.921 (1) Å) due to the Jahn-Teller effect of Mn(III) ion.

In most of phenoxyacetylhydrazone complexes, the phenoxy oxygen atom is not coordinated to metal atom. This structural phenomenon is found in the title complex (I) and was also found in several known complexes (Chen & Liu, 2004; Sun *et al.*, 2005; Chen & Liu, 2006).

The complex Co(C_2_H_3_O_2_)(C_4_H_9_NO)_2_(C_15_H_11_N_3_O_5_) (II) (Sun *et al.*, 2005) and the title complex Mn(C_15_H_11_N_3_O_5_)(C_5_H_7_O_2_)(H_2_O) (I) have the same *N*-salicylaldehyde-N'– (*o*-nitrophenoxyacyl) hydrazone ligand. The dihedral angles between the two benzene rings of the phenoxyacyl hydrazone ligand in complex (I) is 89.48 (8)°, while the corresponding one in (II) is 44.1 (1)°. This big difference of the two dihedral angles may come from the different sizes of the second ligands in the two complexes.

As shown in Fig. 2, two neighboring complex molecules are linked by four hydrogen bonds O—H(coordinated water molecule)···*O*(carbonyl O or oxygen in *o*-nitrate group) to form a centrosymmetrical dimer.

### Experimental

The *N*-salicylaldehyde-*N'*- (*o*-nitrophenoxyacyl) hydrazone ligand, (H_2_L) was prepared according the reference (Sun *et al.*, 2005). H_2_L (0.05 mmol) and Mn(acac)_3_ (0.05 mmol) were dissolved in a mixed solution of 10 ml 95% EtOH and 1 ml DMF. The mixture was stirred for 3 h. After one week, Dark-brown crystals of (I) were obtained.

### Refinement

The water H atoms (H01 and H02) were located from the difference Fourier map and refined isotropically, with O—H distance restraints of 0.88 Å. The other H atoms were positioned geometrically, with C—H = 0.93, 0.97 and 0.96 Å for aromatic, methylene and methyl H, respectively, and constrained to ride on their parent atoms, with *U*_iso_(H) = *xU*_eq_(C), where *x* = 1.5 for methyl H and *x* = 1.2 for all other H atoms.

### Crystal data

**Table:**

[Mn(C_15_H_11_N_3_O_5_)(C_5_H_7_O_2_)(H_2_O)]	*F*(000) = 1000
*M**_r_* = 485.33	*D*_x_ = 1.542 Mg m^−^^3^
Monoclinic, *P*2_1_/*c*	Mo *K*α radiation, λ = 0.71073 Å
Hall symbol: -P 2ybc	Cell parameters from 4921 reflections
*a* = 8.5217 (6) Å	θ = 2.3–27.5°
*b* = 13.9263 (10) Å	µ = 0.69 mm^−^^1^
*c* = 17.6311 (10) Å	*T* = 293 K
β = 92.725 (3)°	Prism, red-black
*V* = 2090.0 (2) Å^3^	0.58 × 0.32 × 0.27 mm
*Z* = 4	

### Data collection

**Table:**

Rigaku R-AXIS RAPID Imaging Plate diffractometer	4735 independent reflections
Radiation source: fine-focus sealed tube	3155 reflections with *I* > 2σ(*I*)
graphite	*R*_int_ = 0.046
ω scans	θ_max_ = 27.5°, θ_min_ = 2.3°
Absorption correction: multi-scan (*TEXRAY*; Molecular Structure Corporation, 1999)	*h* = 0→11
*T*_min_ = 0.766, *T*_max_ = 0.832	*k* = 0→18
4735 measured reflections	*l* = −22→22

### Refinement

**Table:**

Refinement on *F*^2^	Primary atom site location: structure-invariant direct methods
Least-squares matrix: full	Secondary atom site location: difference Fourier map
*R*[*F*^2^ > 2σ(*F*^2^)] = 0.038	Hydrogen site location: inferred from neighbouring sites
*wR*(*F*^2^) = 0.086	H-atom parameters constrained
*S* = 0.88	*w* = 1/[σ^2^(*F*_o_^2^) + (0.042*P*)^2^] where *P* = (*F*_o_^2^ + 2*F*_c_^2^)/3
4735 reflections	(Δ/σ)_max_ < 0.001
293 parameters	Δρ_max_ = 0.41 e Å^−^^3^
2 restraints	Δρ_min_ = −0.26 e Å^−^^3^

### Special details

**Table:**

Geometry. All e.s.d.'s (except the e.s.d. in the dihedral angle between two l.s. planes) are estimated using the full covariance matrix. The cell e.s.d.'s are taken into account individually in the estimation of e.s.d.'s in distances, angles and torsion angles; correlations between e.s.d.'s in cell parameters are only used when they are defined by crystal symmetry. An approximate (isotropic) treatment of cell e.s.d.'s is used for estimating e.s.d.'s involving l.s. planes.
Refinement. Refinement of *F*^2^ against ALL reflections. The weighted *R*-factor *wR* and goodness of fit *S* are based on *F*^2^, conventional *R*-factors *R* are based on *F*, with *F* set to zero for negative *F*^2^. The threshold expression of *F*^2^ > σ(*F*^2^) is used only for calculating *R*-factors(gt) *etc*. and is not relevant to the choice of reflections for refinement. *R*-factors based on *F*^2^ are statistically about twice as large as those based on *F*, and *R*- factors based on ALL data will be even larger.

### Fractional atomic coordinates and isotropic or equivalent isotropic displacement parameters (Å^2^)

**Table:**

	*x*	*y*	*z*	*U*_iso_*/*U*_eq_	
Mn1	0.42210 (3)	0.18422 (2)	0.481380 (18)	0.03308 (10)	
N1	0.57698 (19)	0.27034 (12)	0.53316 (9)	0.0331 (4)	
N2	0.73611 (19)	0.24037 (13)	0.53073 (10)	0.0375 (4)	
N3	0.8041 (2)	0.02849 (16)	0.26015 (12)	0.0533 (5)	
O1	0.25674 (16)	0.25554 (11)	0.52037 (9)	0.0418 (4)	
O2	0.61556 (15)	0.11671 (10)	0.46352 (8)	0.0378 (3)	
O3	0.90604 (16)	0.07570 (10)	0.41027 (8)	0.0388 (3)	
O4	0.8243 (2)	−0.04084 (13)	0.30114 (10)	0.0592 (5)	
O5	0.7271 (3)	0.02349 (18)	0.20070 (14)	0.1154 (10)	
O6	0.44030 (18)	0.26593 (11)	0.38003 (9)	0.0456 (4)	
O7	0.29877 (16)	0.09269 (10)	0.42284 (8)	0.0378 (3)	
O1W	0.39896 (18)	0.07903 (11)	0.57926 (8)	0.0445 (4)	
H01	0.3227	0.0898	0.6102	0.084 (10)*	
H02	0.3985	0.0165	0.5720	0.059 (8)*	
C1	0.2598 (2)	0.34515 (15)	0.54655 (12)	0.0363 (5)	
C2	0.3993 (2)	0.39648 (15)	0.56558 (11)	0.0356 (5)	
C3	0.3901 (3)	0.49157 (16)	0.59191 (12)	0.0447 (6)	
H3A	0.4822	0.5254	0.6036	0.054*	
C4	0.2482 (3)	0.53520 (17)	0.60058 (14)	0.0545 (6)	
H4A	0.2434	0.5984	0.6173	0.065*	
C5	0.1116 (3)	0.48358 (19)	0.58401 (14)	0.0547 (7)	
H5A	0.0149	0.5125	0.5907	0.066*	
C6	0.1161 (3)	0.39072 (17)	0.55798 (13)	0.0474 (6)	
H6A	0.0226	0.3576	0.5478	0.057*	
C7	0.5521 (2)	0.35502 (15)	0.55894 (11)	0.0364 (5)	
H7A	0.6389	0.3919	0.5743	0.044*	
C8	0.7392 (2)	0.16010 (15)	0.49417 (12)	0.0342 (5)	
C9	0.8935 (2)	0.10965 (16)	0.48634 (12)	0.0398 (5)	
H9A	0.9791	0.1536	0.4990	0.048*	
H9B	0.9013	0.0559	0.5213	0.048*	
C10	0.9278 (2)	0.14196 (15)	0.35479 (12)	0.0361 (5)	
C11	0.8798 (2)	0.11932 (16)	0.27964 (13)	0.0406 (5)	
C12	0.9049 (3)	0.18249 (19)	0.22091 (14)	0.0531 (6)	
H12A	0.8707	0.1669	0.1716	0.064*	
C13	0.9798 (3)	0.26774 (18)	0.23514 (16)	0.0570 (7)	
H13A	0.9990	0.3095	0.1955	0.068*	
C14	1.0266 (3)	0.29151 (18)	0.30817 (16)	0.0552 (7)	
H14A	1.0773	0.3497	0.3177	0.066*	
C15	0.9996 (3)	0.23052 (17)	0.36756 (13)	0.0457 (6)	
H15A	1.0297	0.2488	0.4169	0.055*	
C16	0.4860 (4)	0.28832 (19)	0.25042 (16)	0.0682 (8)	
H16A	0.4550	0.3539	0.2572	0.102*	
H16B	0.4391	0.2643	0.2036	0.102*	
H16C	0.5983	0.2848	0.2489	0.102*	
C17	0.4325 (3)	0.22903 (17)	0.31512 (13)	0.0446 (5)	
C18	0.3747 (3)	0.13544 (18)	0.29967 (13)	0.0479 (6)	
H18A	0.3812	0.1129	0.2503	0.057*	
C19	0.3099 (2)	0.07523 (16)	0.35096 (12)	0.0378 (5)	
C20	0.2423 (3)	−0.01982 (16)	0.32597 (13)	0.0471 (6)	
H20A	0.1363	−0.0249	0.3417	0.071*	
H20B	0.3044	−0.0707	0.3486	0.071*	
H20C	0.2430	−0.0246	0.2717	0.071*	

### Atomic displacement parameters (Å^2^)

**Table:**

	*U*^11^	*U*^22^	*U*^33^	*U*^12^	*U*^13^	*U*^23^
Mn1	0.03206 (16)	0.03084 (17)	0.03640 (18)	0.00197 (14)	0.00244 (12)	−0.00560 (15)
N1	0.0330 (9)	0.0332 (10)	0.0331 (9)	0.0030 (7)	0.0021 (8)	−0.0026 (8)
N2	0.0315 (9)	0.0399 (11)	0.0413 (10)	0.0049 (8)	0.0021 (8)	−0.0051 (8)
N3	0.0568 (12)	0.0530 (14)	0.0498 (13)	−0.0075 (11)	−0.0001 (11)	−0.0056 (11)
O1	0.0340 (8)	0.0387 (8)	0.0533 (9)	0.0027 (6)	0.0078 (7)	−0.0123 (7)
O2	0.0343 (7)	0.0318 (8)	0.0475 (9)	0.0009 (6)	0.0037 (7)	−0.0063 (7)
O3	0.0420 (8)	0.0379 (8)	0.0371 (8)	0.0071 (7)	0.0087 (7)	−0.0008 (7)
O4	0.0740 (12)	0.0437 (10)	0.0604 (11)	−0.0057 (9)	0.0091 (9)	−0.0050 (9)
O5	0.158 (2)	0.0944 (18)	0.0865 (16)	−0.0486 (17)	−0.0697 (17)	0.0141 (13)
O6	0.0578 (10)	0.0365 (9)	0.0425 (9)	−0.0007 (7)	0.0016 (8)	0.0018 (7)
O7	0.0405 (8)	0.0375 (8)	0.0353 (8)	−0.0033 (6)	0.0028 (7)	−0.0057 (6)
O1W	0.0534 (9)	0.0368 (9)	0.0437 (9)	0.0019 (7)	0.0092 (8)	−0.0013 (7)
C1	0.0419 (11)	0.0358 (12)	0.0314 (11)	0.0069 (9)	0.0029 (9)	−0.0015 (9)
C2	0.0431 (12)	0.0333 (11)	0.0308 (11)	0.0058 (9)	0.0046 (9)	−0.0037 (9)
C3	0.0544 (14)	0.0377 (13)	0.0417 (13)	0.0053 (11)	0.0009 (11)	−0.0045 (10)
C4	0.0717 (17)	0.0369 (13)	0.0547 (15)	0.0181 (13)	0.0010 (13)	−0.0090 (11)
C5	0.0528 (15)	0.0583 (16)	0.0531 (15)	0.0241 (13)	0.0040 (12)	−0.0089 (13)
C6	0.0435 (12)	0.0493 (14)	0.0496 (14)	0.0105 (11)	0.0046 (11)	−0.0072 (12)
C7	0.0390 (11)	0.0357 (12)	0.0345 (12)	−0.0007 (9)	0.0009 (9)	−0.0056 (10)
C8	0.0368 (11)	0.0346 (12)	0.0317 (11)	0.0044 (9)	0.0055 (9)	0.0018 (9)
C9	0.0390 (11)	0.0450 (13)	0.0356 (11)	0.0108 (10)	0.0033 (10)	0.0003 (10)
C10	0.0312 (10)	0.0357 (12)	0.0418 (12)	0.0075 (9)	0.0060 (9)	0.0010 (10)
C11	0.0383 (11)	0.0377 (12)	0.0458 (13)	0.0023 (10)	0.0029 (10)	0.0002 (11)
C12	0.0585 (15)	0.0573 (16)	0.0432 (13)	0.0045 (13)	0.0004 (12)	0.0036 (12)
C13	0.0642 (16)	0.0482 (16)	0.0591 (17)	0.0034 (13)	0.0090 (14)	0.0146 (13)
C14	0.0601 (15)	0.0359 (13)	0.0701 (18)	−0.0029 (11)	0.0095 (14)	−0.0009 (12)
C15	0.0488 (13)	0.0409 (14)	0.0476 (14)	0.0043 (11)	0.0052 (11)	−0.0057 (11)
C16	0.096 (2)	0.0565 (17)	0.0538 (16)	0.0007 (16)	0.0162 (15)	0.0136 (14)
C17	0.0465 (13)	0.0467 (14)	0.0407 (13)	0.0076 (11)	0.0050 (11)	0.0033 (11)
C18	0.0598 (15)	0.0477 (14)	0.0368 (12)	0.0032 (12)	0.0082 (11)	−0.0041 (11)
C19	0.0361 (11)	0.0377 (12)	0.0394 (12)	0.0066 (9)	−0.0007 (10)	−0.0084 (10)
C20	0.0523 (14)	0.0431 (14)	0.0456 (13)	0.0005 (11)	0.0000 (11)	−0.0126 (11)

### Geometric parameters (Å, °)

**Table:**

Mn1—O1	1.8806 (14)	C5—C6	1.373 (3)
Mn1—O7	1.9214 (14)	C5—H5A	0.9300
Mn1—O2	1.9366 (13)	C6—H6A	0.9300
Mn1—N1	1.9746 (16)	C7—H7A	0.9300
Mn1—O6	2.1303 (15)	C8—C9	1.503 (3)
Mn1—O1W	2.2793 (15)	C9—H9A	0.9700
N1—C7	1.285 (3)	C9—H9B	0.9700
N1—N2	1.421 (2)	C10—C15	1.390 (3)
N2—C8	1.291 (3)	C10—C11	1.404 (3)
N3—O5	1.212 (3)	C11—C12	1.383 (3)
N3—O4	1.213 (2)	C12—C13	1.366 (4)
N3—C11	1.454 (3)	C12—H12A	0.9300
O1—C1	1.330 (2)	C13—C14	1.370 (4)
O2—C8	1.309 (2)	C13—H13A	0.9300
O3—C10	1.364 (2)	C14—C15	1.376 (3)
O3—C9	1.431 (2)	C14—H14A	0.9300
O6—C17	1.253 (3)	C15—H15A	0.9300
O7—C19	1.298 (2)	C16—C17	1.497 (3)
O1W—H01	0.8802	C16—H16A	0.9600
O1W—H02	0.8799	C16—H16B	0.9600
C1—C6	1.402 (3)	C16—H16C	0.9600
C1—C2	1.414 (3)	C17—C18	1.415 (3)
C2—C3	1.407 (3)	C18—C19	1.368 (3)
C2—C7	1.435 (3)	C18—H18A	0.9300
C3—C4	1.368 (3)	C19—C20	1.501 (3)
C3—H3A	0.9300	C20—H20A	0.9600
C4—C5	1.387 (4)	C20—H20B	0.9600
C4—H4A	0.9300	C20—H20C	0.9600
			
O1—Mn1—O7	98.43 (6)	C2—C7—H7A	117.8
O1—Mn1—O2	166.98 (7)	N2—C8—O2	124.81 (18)
O7—Mn1—O2	92.22 (6)	N2—C8—C9	119.28 (19)
O1—Mn1—N1	90.36 (7)	O2—C8—C9	115.90 (18)
O7—Mn1—N1	171.05 (6)	O3—C9—C8	110.20 (16)
O2—Mn1—N1	79.31 (6)	O3—C9—H9A	109.6
O1—Mn1—O6	96.33 (7)	C8—C9—H9A	109.6
O7—Mn1—O6	87.91 (6)	O3—C9—H9B	109.6
O2—Mn1—O6	91.51 (6)	C8—C9—H9B	109.6
N1—Mn1—O6	89.40 (6)	H9A—C9—H9B	108.1
O1—Mn1—O1W	88.25 (6)	O3—C10—C15	123.9 (2)
O7—Mn1—O1W	85.15 (6)	O3—C10—C11	118.78 (19)
O2—Mn1—O1W	85.19 (6)	C15—C10—C11	117.3 (2)
N1—Mn1—O1W	96.91 (6)	C12—C11—C10	121.0 (2)
O6—Mn1—O1W	172.19 (6)	C12—C11—N3	117.3 (2)
C7—N1—N2	116.98 (17)	C10—C11—N3	121.6 (2)
C7—N1—Mn1	127.08 (14)	C13—C12—C11	120.2 (2)
N2—N1—Mn1	115.18 (12)	C13—C12—H12A	119.9
C8—N2—N1	108.13 (16)	C11—C12—H12A	119.9
O5—N3—O4	121.7 (2)	C12—C13—C14	119.7 (2)
O5—N3—C11	118.1 (2)	C12—C13—H13A	120.2
O4—N3—C11	120.2 (2)	C14—C13—H13A	120.2
C1—O1—Mn1	128.29 (13)	C13—C14—C15	120.9 (2)
C8—O2—Mn1	112.55 (12)	C13—C14—H14A	119.5
C10—O3—C9	117.88 (17)	C15—C14—H14A	119.5
C17—O6—Mn1	122.95 (15)	C14—C15—C10	120.8 (2)
C19—O7—Mn1	125.80 (13)	C14—C15—H15A	119.6
Mn1—O1W—H01	116.9	C10—C15—H15A	119.6
Mn1—O1W—H02	121.7	C17—C16—H16A	109.5
H01—O1W—H02	105.0	C17—C16—H16B	109.5
O1—C1—C6	118.1 (2)	H16A—C16—H16B	109.5
O1—C1—C2	123.98 (18)	C17—C16—H16C	109.5
C6—C1—C2	117.85 (19)	H16A—C16—H16C	109.5
C3—C2—C1	119.68 (19)	H16B—C16—H16C	109.5
C3—C2—C7	118.1 (2)	O6—C17—C18	123.8 (2)
C1—C2—C7	122.25 (18)	O6—C17—C16	117.7 (2)
C4—C3—C2	121.2 (2)	C18—C17—C16	118.5 (2)
C4—C3—H3A	119.4	C19—C18—C17	125.8 (2)
C2—C3—H3A	119.4	C19—C18—H18A	117.1
C3—C4—C5	118.9 (2)	C17—C18—H18A	117.1
C3—C4—H4A	120.5	O7—C19—C18	125.5 (2)
C5—C4—H4A	120.5	O7—C19—C20	113.99 (19)
C6—C5—C4	121.5 (2)	C18—C19—C20	120.5 (2)
C6—C5—H5A	119.3	C19—C20—H20A	109.5
C4—C5—H5A	119.3	C19—C20—H20B	109.5
C5—C6—C1	120.8 (2)	H20A—C20—H20B	109.5
C5—C6—H6A	119.6	C19—C20—H20C	109.5
C1—C6—H6A	119.6	H20A—C20—H20C	109.5
N1—C7—C2	124.32 (19)	H20B—C20—H20C	109.5
N1—C7—H7A	117.8		
			
O1—Mn1—N1—C7	−18.44 (18)	O1—C1—C6—C5	−179.2 (2)
O2—Mn1—N1—C7	169.54 (18)	C2—C1—C6—C5	2.5 (3)
O6—Mn1—N1—C7	77.89 (17)	N2—N1—C7—C2	−179.77 (19)
O1W—Mn1—N1—C7	−106.72 (17)	Mn1—N1—C7—C2	10.7 (3)
O1—Mn1—N1—N2	171.88 (14)	C3—C2—C7—N1	−177.42 (19)
O2—Mn1—N1—N2	−0.15 (13)	C1—C2—C7—N1	2.5 (3)
O6—Mn1—N1—N2	−91.80 (14)	N1—N2—C8—O2	1.6 (3)
O1W—Mn1—N1—N2	83.60 (13)	N1—N2—C8—C9	−177.13 (17)
C7—N1—N2—C8	−171.41 (17)	Mn1—O2—C8—N2	−1.8 (3)
Mn1—N1—N2—C8	−0.6 (2)	Mn1—O2—C8—C9	176.98 (14)
O7—Mn1—O1—C1	−157.85 (17)	C10—O3—C9—C8	71.6 (2)
O2—Mn1—O1—C1	57.6 (4)	N2—C8—C9—O3	−136.04 (19)
N1—Mn1—O1—C1	20.41 (17)	O2—C8—C9—O3	45.1 (2)
O6—Mn1—O1—C1	−69.03 (17)	C9—O3—C10—C15	27.0 (3)
O1W—Mn1—O1—C1	117.32 (17)	C9—O3—C10—C11	−155.01 (18)
O1—Mn1—O2—C8	−37.1 (3)	O3—C10—C11—C12	−177.42 (19)
O7—Mn1—O2—C8	177.99 (13)	C15—C10—C11—C12	0.7 (3)
N1—Mn1—O2—C8	0.91 (13)	O3—C10—C11—N3	1.6 (3)
O6—Mn1—O2—C8	90.02 (14)	C15—C10—C11—N3	179.81 (19)
O1W—Mn1—O2—C8	−97.07 (13)	O5—N3—C11—C12	−21.3 (4)
O1—Mn1—O6—C17	−125.31 (18)	O4—N3—C11—C12	155.4 (2)
O7—Mn1—O6—C17	−27.06 (18)	O5—N3—C11—C10	159.6 (2)
O2—Mn1—O6—C17	65.11 (18)	O4—N3—C11—C10	−23.7 (3)
N1—Mn1—O6—C17	144.40 (18)	C10—C11—C12—C13	1.1 (4)
O1—Mn1—O7—C19	125.06 (16)	N3—C11—C12—C13	−178.0 (2)
O2—Mn1—O7—C19	−62.46 (16)	C11—C12—C13—C14	−1.6 (4)
O6—Mn1—O7—C19	28.97 (16)	C12—C13—C14—C15	0.2 (4)
O1W—Mn1—O7—C19	−147.44 (16)	C13—C14—C15—C10	1.7 (4)
Mn1—O1—C1—C6	166.84 (16)	O3—C10—C15—C14	175.9 (2)
Mn1—O1—C1—C2	−15.0 (3)	C11—C10—C15—C14	−2.2 (3)
O1—C1—C2—C3	179.15 (19)	Mn1—O6—C17—C18	15.6 (3)
C6—C1—C2—C3	−2.6 (3)	Mn1—O6—C17—C16	−165.12 (17)
O1—C1—C2—C7	−0.8 (3)	O6—C17—C18—C19	4.8 (4)
C6—C1—C2—C7	177.4 (2)	C16—C17—C18—C19	−174.5 (2)
C1—C2—C3—C4	1.0 (3)	Mn1—O7—C19—C18	−20.4 (3)
C7—C2—C3—C4	−179.1 (2)	Mn1—O7—C19—C20	160.21 (14)
C2—C3—C4—C5	1.0 (4)	C17—C18—C19—O7	−3.8 (4)
C3—C4—C5—C6	−1.1 (4)	C17—C18—C19—C20	175.6 (2)
C4—C5—C6—C1	−0.6 (4)		

### Hydrogen-bond geometry (Å, °)

**Table:**

*D*—H···*A*	*D*—H	H···*A*	*D*···*A*	*D*—H···*A*
O1W—H01···O4^i^	0.88	2.16	2.955 (2)	150
O1W—H02···O2^i^	0.88	1.96	2.829 (2)	169

Symmetry codes: (i) −*x*+1, −*y*, −*z*+1.
